# 2-{(*E*)-[(4-Methyl­phen­yl)imino]­meth­yl}-4-nitro­phenol–2-{(*E*)-[(4-methyl­phen­yl)iminio]meth­yl}-4-nitro­phenolate (0.60/0.40)

**DOI:** 10.1107/S1600536811032028

**Published:** 2011-08-11

**Authors:** M. Nawaz Tahir, Hazoor Ahmad Shad, Riaz H. Tariq

**Affiliations:** a Department of Physics, University of Sargodha, Sargodha, Pakistan; b Department of Chemistry, Govt. M. D. College, Toba Tek Singh, Punjab, Pakistan; c Institute of Chemical and Pharmaceutical Sciences, The University of Faisalabad, Faisalabad, Pakistan

## Abstract

The crystal of the title compound, 0.6C_14_H_12_N_2_O_3_·0.4C_14_H_12_N_2_O_3_, contains a mixture of its neutral (OH containing) and zwitterionic (NH containing) forms, in a 0.60 (4):0.40 (4) ratio. The former generates an *S*(6) loop *via* an intra­molecular O—H⋯N hydrogen bond and the latter generates an *S*(6) loop *via* an N—H⋯O hydro­gren bond. The aromatic rings are oriented at a dihedral angle of 42.52 (10)°. In the crystal, C—H⋯π inter­actions occur and aromatic π–π stacking inter­actions [centroid–centroid separations = 3.7106 (12) and 3.9177 (13) Å] consolidate the packing.

## Related literature

For related structures, see: Hijji *et al.* (2009 ▶); Kılıç *et al.* (2009 ▶). For graph-set notation, see: Bernstein *et al.* (1995 ▶).
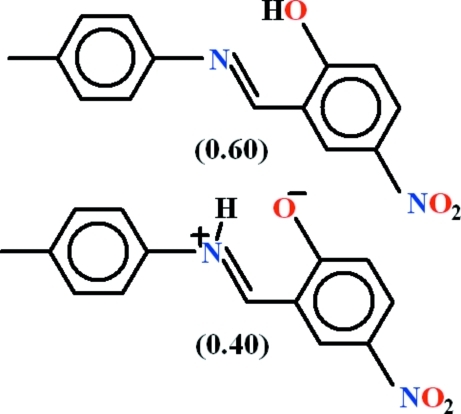


## Experimental

### 

#### Crystal data

0.6C_14_H_12_N_2_O_3_·0.4C_14_H_12_N_2_O_3_
*M* *_r_* = 256.26
Monoclinic, 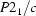

*a* = 14.0623 (6) Å
*b* = 14.1723 (8) Å
*c* = 6.2357 (3) Å
β = 95.400 (2)°
*V* = 1237.23 (11) Å^3^
*Z* = 4
Mo *K*α radiation
μ = 0.10 mm^−1^
*T* = 296 K
0.25 × 0.22 × 0.20 mm

#### Data collection

Bruker Kappa APEXII CCD diffractometer
Absorption correction: multi-scan (*SADABS*; Bruker, 2005 ▶) *T*_min_ = 0.976, *T*_max_ = 0.985
9733 measured reflections
2238 independent reflections
1395 reflections with *I* > 2σ(*I*)
*R*_int_ = 0.041

#### Refinement

*R*[*F* ^2^ > 2σ(*F* ^2^)] = 0.049
*wR*(*F* ^2^) = 0.133
*S* = 1.03
2238 reflections
181 parameters
H atoms treated by a mixture of independent and constrained refinement
Δρ_max_ = 0.20 e Å^−3^
Δρ_min_ = −0.18 e Å^−3^

### 

Data collection: *APEX2* (Bruker, 2009 ▶); cell refinement: *SAINT* (Bruker, 2009 ▶); data reduction: *SAINT* (Bruker, 2009 ▶); program(s) used to solve structure: *SHELXS97* (Sheldrick, 2008 ▶); program(s) used to refine structure: *SHELXL97* (Sheldrick, 2008 ▶); molecular graphics: *ORTEP-3 for Windows* (Farrugia, 1997 ▶) and *PLATON* (Spek, 2009 ▶); software used to prepare material for publication: *WinGX* (Farrugia, 1999 ▶) and *PLATON* (Spek, 2009 ▶).

## Supplementary Material

Crystal structure: contains datablock(s) global, I. DOI: 10.1107/S1600536811032028/hb6352sup1.cif

Structure factors: contains datablock(s) I. DOI: 10.1107/S1600536811032028/hb6352Isup2.hkl

Supplementary material file. DOI: 10.1107/S1600536811032028/hb6352Isup3.cml

Additional supplementary materials:  crystallographic information; 3D view; checkCIF report

## Figures and Tables

**Table 1:** Hydrogen-bond geometry (Å, °) *Cg*1 and *Cg*2 are the centroids of the C1—C6 and C9—C14 rings, respectively.

*D*—H⋯*A*	*D*—H	H⋯*A*	*D*⋯*A*	*D*—H⋯*A*
O1—H1⋯N1	0.80 (6)	1.86 (6)	2.566 (3)	147 (6)
N1—H1*A*⋯O1	0.97 (8)	1.71 (7)	2.566 (3)	146 (6)
C5—H5⋯*Cg*1^i^	0.93	2.84	3.515 (2)	130
C11—H11⋯*Cg*2^ii^	0.93	2.82	3.490 (2)	130

Symmetry codes: (i) 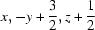; (ii) 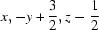.

**Fig. 1.**
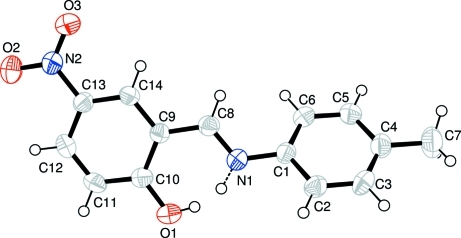
View of the title compound with displacement ellipsoids drawn at the 50% probability level. The dotted line represents the miniority H-atom.

**Fig. 2.**
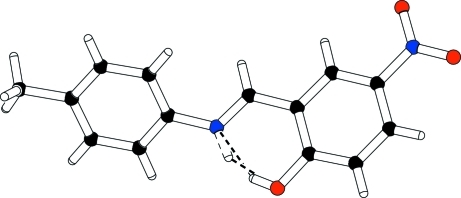
Overlapped intramolecular hydrogen bonds forming two S(6) ring motif.

## supplementary crystallographic information

### Comment

The crystal structures of 2-((4-methoxyphenyl)iminomethyl)-4-nitrophenol (Kılıç *et al.*, 2009) and 2-(((*E*)-(2-hydroxy-5-nitrophenyl) methylidene)ammonio)-4-nitrophenolate (Hijji *et al.*, 2009) have been published which are related to the title compound (I), (Fig. 1). The title compound consists of two isomers with 0.60:0.40 ratio.

In (I), the 4-methylanilinic group A (C1—C7/N1) and the 2-hydroxy-5-nitrobenzaldehyde group B (C8—C14/N2/O1/O2/O3) are almost planar with r.m.s. deviations of 0.0283 and 0.0222 Å, respectively. The dihedral angle between A/B is 41.86 (4)°. There exist intramolecular hydrogen bonds of O—H···N and N—H···O type completing S(6) (Bernstein *et al.*, 1995) ring motifs (Table 1, Fig. 2). In the crystal, there exist π–π interaction between the centroids of the benzene rings of 4-methyaniline with a separation of 3.9177 (13) Å and a slippage of 1.333 Å. Similarly, π–π interaction between the centroids of the benzene rings of 2-hydroxy-5-nitrobenzaldehyde also exist with a separation of 3.7106 (12) Å and a slippage of 1.452 Å. In consolidating the crystal structure, C—H···π bonds (Table 1) also play role.

### Experimental

Equimolar quantities of 2-hydroxy-5-nitrobenzaldehyde and and 4-methylaniline were refluxed in ethanol for 30 min resulting in a yellow solution. The solution was kept at room temperature which affoarded yellow prisms of (I) after three days.

### Refinement

The coordinates of hydrogen atoms of O—H and N—H were refined. The occupancy factor of both H-atoms was refined and therefore, the two isomers are of 0.60:0.40 ratio. The H-atoms were positioned geometrically (C–H = 0.93 Å) and refined as riding with *U*_iso_(H) = *xU*_eq_(C, N, O), where *x* = 1.2 for all H-atoms.

### Crystal data

**Table:**

0.6C_14_H_12_N_2_O_3_·0.4C_14_H_12_N_2_O_3_	*F*(000) = 536
*M**_r_* = 256.26	*D*_x_ = 1.376 Mg m^−^^3^
Monoclinic, *P*2_1_/*c*	Mo *K*α radiation, λ = 0.71073 Å
Hall symbol: -P 2ybc	Cell parameters from 1395 reflections
*a* = 14.0623 (6) Å	θ = 2.0–25.3°
*b* = 14.1723 (8) Å	µ = 0.10 mm^−^^1^
*c* = 6.2357 (3) Å	*T* = 296 K
β = 95.400 (2)°	Prism, yellow
*V* = 1237.23 (11) Å^3^	0.25 × 0.22 × 0.20 mm
*Z* = 4	

### Data collection

**Table:**

Bruker Kappa APEXII CCD diffractometer	2238 independent reflections
Radiation source: fine-focus sealed tube	1395 reflections with *I* > 2σ(*I*)
graphite	*R*_int_ = 0.041
Detector resolution: 8.10 pixels mm^-1^	θ_max_ = 25.3°, θ_min_ = 2.0°
ω scans	*h* = −16→16
Absorption correction: multi-scan (*SADABS*; Bruker, 2005)	*k* = −17→17
*T*_min_ = 0.976, *T*_max_ = 0.985	*l* = −7→7
9733 measured reflections	

### Refinement

**Table:**

Refinement on *F*^2^	Secondary atom site location: difference Fourier map
Least-squares matrix: full	Hydrogen site location: inferred from neighbouring sites
*R*[*F*^2^ > 2σ(*F*^2^)] = 0.049	H atoms treated by a mixture of independent and constrained refinement
*wR*(*F*^2^) = 0.133	*w* = 1/[σ^2^(*F*_o_^2^) + (0.0558*P*)^2^ + 0.2667*P*] where *P* = (*F*_o_^2^ + 2*F*_c_^2^)/3
*S* = 1.03	(Δ/σ)_max_ < 0.001
2238 reflections	Δρ_max_ = 0.20 e Å^−^^3^
181 parameters	Δρ_min_ = −0.18 e Å^−^^3^
0 restraints	Extinction correction: *SHELXL97* (Sheldrick, 2008), Fc^*^=kFc[1+0.001xFc^2^λ^3^/sin(2θ)]^-1/4^
Primary atom site location: structure-invariant direct methods	Extinction coefficient: 0.0052 (14)

### Special details

**Table:**

Geometry. Bond distances, angles *etc*. have been calculated using the rounded fractional coordinates. All su's are estimated from the variances of the (full) variance-covariance matrix. The cell e.s.d.'s are taken into account in the estimation of distances, angles and torsion angles
Refinement. Refinement of *F*^2^ against ALL reflections. The weighted *R*-factor *wR* and goodness of fit *S* are based on *F*^2^, conventional *R*-factors *R* are based on *F*, with *F* set to zero for negative *F*^2^. The threshold expression of *F*^2^ > σ(*F*^2^) is used only for calculating *R*-factors(gt) *etc*. and is not relevant to the choice of reflections for refinement. *R*-factors based on *F*^2^ are statistically about twice as large as those based on *F*, and *R*- factors based on ALL data will be even larger.

### Fractional atomic coordinates and isotropic or equivalent isotropic displacement parameters (Å^2^)

**Table:**

	*x*	*y*	*z*	*U*_iso_*/*U*_eq_	Occ. (<1)
O1	0.18732 (12)	0.66637 (13)	0.3141 (3)	0.0536 (6)	
O2	−0.21627 (11)	0.59616 (18)	0.6085 (3)	0.0928 (9)	
O3	−0.13153 (11)	0.55621 (14)	0.8965 (3)	0.0676 (7)	
N1	0.28626 (12)	0.62025 (13)	0.6653 (3)	0.0406 (6)	
N2	−0.13922 (12)	0.58577 (15)	0.7115 (3)	0.0499 (7)	
C1	0.37651 (14)	0.62162 (15)	0.7884 (3)	0.0379 (7)	
C2	0.45601 (14)	0.59352 (16)	0.6902 (4)	0.0452 (8)	
C3	0.54536 (15)	0.59847 (17)	0.8003 (4)	0.0507 (9)	
C4	0.55810 (15)	0.63374 (16)	1.0085 (4)	0.0465 (8)	
C5	0.47796 (15)	0.66189 (16)	1.1032 (3)	0.0453 (8)	
C6	0.38783 (14)	0.65645 (16)	0.9965 (3)	0.0429 (8)	
C7	0.65595 (16)	0.6427 (2)	1.1264 (4)	0.0708 (10)	
C8	0.20829 (14)	0.60487 (15)	0.7508 (3)	0.0394 (7)	
C9	0.11702 (14)	0.61460 (15)	0.6278 (3)	0.0353 (7)	
C10	0.11017 (15)	0.64786 (15)	0.4120 (3)	0.0392 (7)	
C11	0.01983 (15)	0.66214 (15)	0.3029 (3)	0.0431 (8)	
C12	−0.06138 (15)	0.64214 (15)	0.3987 (3)	0.0431 (8)	
C13	−0.05334 (14)	0.60742 (15)	0.6096 (3)	0.0382 (7)	
C14	0.03375 (13)	0.59379 (15)	0.7226 (3)	0.0365 (7)	
H1	0.234 (4)	0.652 (3)	0.390 (10)	0.0643*	0.60 (4)
H2	0.44905	0.57122	0.54928	0.0542*	
H3	0.59813	0.57782	0.73398	0.0608*	
H5	0.48500	0.68523	1.24322	0.0544*	
H6	0.33496	0.67605	1.06375	0.0515*	
H7A	0.65363	0.62414	1.27390	0.1061*	
H7B	0.67695	0.70705	1.12087	0.1061*	
H7C	0.69974	0.60270	1.05983	0.1061*	
H8	0.21077	0.58713	0.89483	0.0472*	
H11	0.01490	0.68561	0.16300	0.0517*	
H12	−0.12117	0.65149	0.32459	0.0517*	
H14	0.03733	0.57065	0.86269	0.0438*	
H1A	0.274 (5)	0.641 (4)	0.518 (13)	0.0487*	0.40 (4)

### Atomic displacement parameters (Å^2^)

**Table:**

	*U*^11^	*U*^22^	*U*^33^	*U*^12^	*U*^13^	*U*^23^
O1	0.0503 (10)	0.0696 (13)	0.0423 (10)	−0.0045 (9)	0.0123 (8)	0.0082 (8)
O2	0.0349 (10)	0.171 (2)	0.0711 (13)	0.0043 (12)	−0.0028 (9)	0.0236 (13)
O3	0.0485 (10)	0.1043 (16)	0.0511 (11)	−0.0035 (9)	0.0110 (8)	0.0189 (10)
N1	0.0362 (10)	0.0452 (12)	0.0407 (11)	0.0013 (8)	0.0053 (8)	0.0002 (9)
N2	0.0361 (10)	0.0681 (14)	0.0454 (12)	−0.0001 (10)	0.0039 (9)	0.0003 (10)
C1	0.0328 (11)	0.0393 (13)	0.0417 (13)	0.0008 (9)	0.0045 (9)	0.0027 (10)
C2	0.0424 (12)	0.0507 (15)	0.0435 (13)	0.0027 (11)	0.0094 (10)	−0.0063 (11)
C3	0.0354 (12)	0.0554 (16)	0.0627 (16)	0.0073 (11)	0.0127 (11)	−0.0019 (12)
C4	0.0390 (12)	0.0473 (15)	0.0524 (14)	0.0023 (10)	0.0008 (10)	0.0044 (12)
C5	0.0457 (13)	0.0513 (15)	0.0391 (12)	0.0015 (11)	0.0044 (10)	−0.0016 (11)
C6	0.0387 (12)	0.0489 (14)	0.0424 (13)	0.0016 (10)	0.0100 (10)	−0.0003 (11)
C7	0.0458 (14)	0.086 (2)	0.0776 (19)	0.0041 (14)	−0.0102 (13)	−0.0008 (16)
C8	0.0398 (12)	0.0403 (14)	0.0383 (12)	0.0014 (10)	0.0053 (10)	0.0032 (10)
C9	0.0350 (11)	0.0356 (12)	0.0351 (12)	−0.0005 (9)	0.0030 (9)	0.0003 (9)
C10	0.0432 (12)	0.0378 (13)	0.0372 (12)	−0.0030 (10)	0.0077 (10)	−0.0014 (10)
C11	0.0537 (14)	0.0440 (14)	0.0309 (11)	−0.0015 (11)	0.0005 (10)	0.0046 (10)
C12	0.0427 (12)	0.0441 (14)	0.0406 (13)	0.0014 (10)	−0.0067 (10)	−0.0023 (10)
C13	0.0351 (11)	0.0422 (14)	0.0378 (12)	−0.0019 (9)	0.0055 (9)	−0.0025 (10)
C14	0.0392 (11)	0.0394 (13)	0.0308 (11)	−0.0002 (10)	0.0029 (9)	0.0007 (9)

### Geometric parameters (Å, °)

**Table:**

O1—C10	1.320 (3)	C9—C10	1.421 (3)
O2—N2	1.215 (2)	C10—C11	1.398 (3)
O3—N2	1.223 (3)	C11—C12	1.367 (3)
O1—H1	0.80 (6)	C12—C13	1.399 (3)
N1—C1	1.420 (3)	C13—C14	1.368 (3)
N1—C8	1.282 (3)	C2—H2	0.9300
N2—C13	1.449 (3)	C3—H3	0.9300
N1—H1A	0.96 (8)	C5—H5	0.9300
C1—C2	1.383 (3)	C6—H6	0.9300
C1—C6	1.384 (3)	C7—H7A	0.9600
C2—C3	1.376 (3)	C7—H7B	0.9600
C3—C4	1.387 (3)	C7—H7C	0.9600
C4—C5	1.380 (3)	C8—H8	0.9300
C4—C7	1.503 (3)	C11—H11	0.9300
C5—C6	1.377 (3)	C12—H12	0.9300
C8—C9	1.439 (3)	C14—H14	0.9300
C9—C14	1.392 (3)		
			
C10—O1—H1	110 (4)	N2—C13—C12	119.32 (18)
C1—N1—C8	122.22 (18)	N2—C13—C14	119.13 (17)
O2—N2—O3	122.38 (18)	C12—C13—C14	121.55 (18)
O3—N2—C13	118.87 (17)	C9—C14—C13	119.96 (18)
O2—N2—C13	118.74 (18)	C1—C2—H2	120.00
C8—N1—H1A	111 (4)	C3—C2—H2	120.00
C1—N1—H1A	126 (4)	C2—C3—H3	119.00
N1—C1—C2	118.38 (18)	C4—C3—H3	119.00
N1—C1—C6	122.00 (18)	C4—C5—H5	119.00
C2—C1—C6	119.45 (19)	C6—C5—H5	119.00
C1—C2—C3	120.2 (2)	C1—C6—H6	120.00
C2—C3—C4	121.1 (2)	C5—C6—H6	120.00
C3—C4—C5	117.8 (2)	C4—C7—H7A	109.00
C3—C4—C7	121.3 (2)	C4—C7—H7B	109.00
C5—C4—C7	120.9 (2)	C4—C7—H7C	109.00
C4—C5—C6	121.92 (19)	H7A—C7—H7B	109.00
C1—C6—C5	119.51 (18)	H7A—C7—H7C	109.00
N1—C8—C9	121.13 (18)	H7B—C7—H7C	109.00
C8—C9—C14	119.82 (17)	N1—C8—H8	119.00
C8—C9—C10	120.96 (18)	C9—C8—H8	119.00
C10—C9—C14	119.20 (18)	C10—C11—H11	119.00
O1—C10—C9	121.23 (19)	C12—C11—H11	119.00
O1—C10—C11	119.66 (18)	C11—C12—H12	120.00
C9—C10—C11	119.11 (18)	C13—C12—H12	120.00
C10—C11—C12	121.02 (18)	C9—C14—H14	120.00
C11—C12—C13	119.13 (19)	C13—C14—H14	120.00
			
C8—N1—C1—C2	149.4 (2)	C4—C5—C6—C1	0.2 (3)
C8—N1—C1—C6	−35.4 (3)	N1—C8—C9—C10	−4.4 (3)
C1—N1—C8—C9	172.72 (19)	N1—C8—C9—C14	177.4 (2)
O2—N2—C13—C12	1.9 (3)	C8—C9—C10—O1	3.4 (3)
O2—N2—C13—C14	−178.5 (2)	C8—C9—C10—C11	−175.9 (2)
O3—N2—C13—C12	−179.4 (2)	C14—C9—C10—O1	−178.4 (2)
O3—N2—C13—C14	0.3 (3)	C14—C9—C10—C11	2.3 (3)
N1—C1—C2—C3	176.6 (2)	C8—C9—C14—C13	176.8 (2)
C6—C1—C2—C3	1.3 (3)	C10—C9—C14—C13	−1.4 (3)
N1—C1—C6—C5	−175.7 (2)	O1—C10—C11—C12	178.9 (2)
C2—C1—C6—C5	−0.5 (3)	C9—C10—C11—C12	−1.8 (3)
C1—C2—C3—C4	−1.7 (4)	C10—C11—C12—C13	0.4 (3)
C2—C3—C4—C5	1.3 (3)	C11—C12—C13—N2	−179.76 (19)
C2—C3—C4—C7	−177.7 (2)	C11—C12—C13—C14	0.6 (3)
C3—C4—C5—C6	−0.6 (3)	N2—C13—C14—C9	−179.70 (19)
C7—C4—C5—C6	178.4 (2)	C12—C13—C14—C9	0.0 (3)

### Hydrogen-bond geometry (Å, °)

**Table:**

Cg1 and Cg2 are the centroids of the C1—C6 and C9—C14 rings, respectively.

**Table:**

*D*—H···*A*	*D*—H	H···*A*	*D*···*A*	*D*—H···*A*
O1—H1···N1	0.80 (6)	1.86 (6)	2.566 (3)	147 (6)
N1—H1A···O1	0.97 (8)	1.71 (7)	2.566 (3)	146 (6)
C5—H5···Cg1^i^	0.93	2.84	3.515 (2)	130
C11—H11···Cg2^ii^	0.93	2.82	3.490 (2)	130

Symmetry codes: (i) *x*, −*y*+3/2, *z*+1/2; (ii) *x*, −*y*+3/2, *z*−1/2.
